# Adenoid cystic carcinoma of the parotid gland: Anastamosis of the facial nerve with the great auricular nerve after radical parotidectomy

**DOI:** 10.4103/0970-0358.44948

**Published:** 2008

**Authors:** Osman Bahadir, Murat Livaoglu, Ahmet Ural

**Affiliations:** Departments of Otorhinolaryngology, Karadeniz Technical, University Faculty of Medicine, Trabzon, TURKEY; 1 Departments of Plastic and Reconstructive Surgery, Karadeniz Technical, University Faculty of Medicine, Trabzon, TURKEY

**Keywords:** Adenoid cystic carcinoma, facial nerve reconstruction, parotid gland, radiotherapy

## Abstract

Adenoid cystic carcinoma of the parotid gland is a rare and slowly growing, but highly malignant tumor. Surgical resection of a malignant parotid tumor should include resection of the facial nerve when the nerve is involved in the tumor. Facial nerve reconstruction is required after nerve resection. A 14 year-old female presented with complaints of painless enlargement of the right parotid gland and facial asymmetry. Physical examination revealed a firm mass in the region of the parotid gland as well as right facial paralysis. Biopsy obtained from the mass showed an adenoid cystic carcinoma of the parotid gland. A radical parotidectomy with a modified radical neck dissection was carried out. Grafting material for the facial reconstruction was harvested from the great auricular nerve. The proximal main trunk and each distal branch of the facial nerve were coapted with the greater auricular nerve. The patient received radiotherapy after surgery and was seen to achieve grade IV facial function one year after surgery. Thus, the great auricular nerve is appropriate grafting material for coaptation of each distal branch of the facial nerve.

## INTRODUCTION

Adenoid cystic carcinomas (ACC) of the head and neck are relatively rare tumors, consisting of approximately 10–15% of all salivary gland neoplasms.[1] They can arise within the major and minor salivary glands or mucous glands of the oral cavity and upper respiratory system. The most common site of occurrence is the palate, followed by the major salivary glands.[2]

The natural history of ACC is characterized by an indolent growth rate, relatively low probability of regional lymph node metastases, and a high likelihood of haematogenous dissemination.[3–5] The most common site of distant metastasis is the lung.[6]

ACC remains an extremely difficult disease to treat. It has a high predisposition for recurrence and metastasis if a patient lives long enough, and this occurs even when radical excision has been performed.[7]

Facial nerve reconstruction is mandatory in malignant tumours of the parotid gland after radical parotidectomy (RP). Although there are many reports of surgical procedures that graft the facial nerve,[8–10] there are still controversies regarding the functional results.

We present here a patient who underwent anastomosis of the main trunk and each distal branch of the facial nerve with the greater auricular nerve (GAN) and also discuss the effect of postoperative radiotherapy.

## CASE REPORT

A 14 year-old female presented with a year's history of painless enlargement of the right parotid gland and one week's history of facial asymmetry. Physical examination revealed a firm mass 2 × 3 cm in diameter in the region of the parotid gland as well as right facial nerve paralysis. The mass was fixed with palpation as was a palpable right cervical lymph node. She had a normal body temperature and normal complete blood count, erythrocyte sedimentation rate, and blood chemistry.

We investigated the possibility of other diseases that could cause a swelling in the parotid gland: granulomatous infections such as mycobacterial diseases and autoimmune disorders. The results were negative.

Magnetic resonance imaging (MRI) showed an enlargement of the right parotid gland with evidence of multiple cystic masses involving both the superficial and deep portions of the gland.

Fine-needle aspiration (FNA) biopsy was used for diagnosis. Cytopathological examination suggested ACC with the finding of large globules of the extracellular matrix, partially surrounded by basaloid tumour cells but lacking characteristic globules.

A radical parotidectomy and modified radical neck dissection with spinal accessory nerve preservation was planned under general anesthesia. A preauricular incision that curved under the ear lobule was made in extension with the neck dissection incision. A skin subcutaneous flap was raised over the parotid until the distal branches of the facial nerve were seen to exit the gland. The mandibular, buccal, and zygomatic branches were incised and tagged with fine silk sutures for later grafting [Figure 1A]. As the main trunk was considered to be encased by the tumour, a mastoidectomy was performed, to identify the main trunk of the facial nerve, increase the length of the proximal nerve stump for grafting, and also to provide adequate surgical margin.[9] Once the main trunk was identified, extensive resection was performed to remove the parotid neoplasm. Frozen sections from the facial nerve confirm uninvolved nerve margins. After resection of the parotid neoplasm, modified neck dissection was subsequently added and the donor nerve graft was prepared from the great auricular nerve [Figure 1B]. The greater auricular nerve was traced proximally until additional branches coming off the cervical nerve roots were isolated.[11] After freshening the cable nerve graft ends as well as the proximal and distal facial nerve branches with a scalpel, microscopic epineural repair was performed with an interrupted 8-0 monofilament nylon suture, with three to five sutures used at each anastomosis. The distal part of the graft was anastomosed to the main trunk of the facial nerve and the proximal branches coming off the cervical nerve roots were anastomosed to peripheral stumps [Figure 1C].

**Figure 1A:**
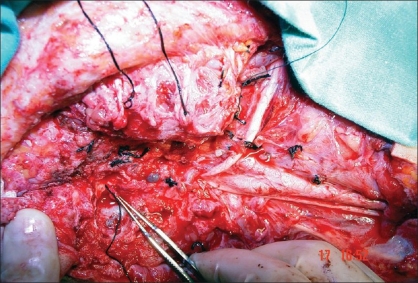
The surgical field after tumor resection shows the proximal main trunk of the facial nerve and the mandibular, buccal, and zygomatic branches to be tagged with fine silk sutures for later grafting

**Figure 1B:**
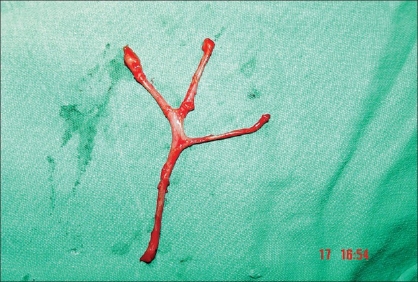
The graft of the great auricular nerve with cervical roots

**Figure 1C:**
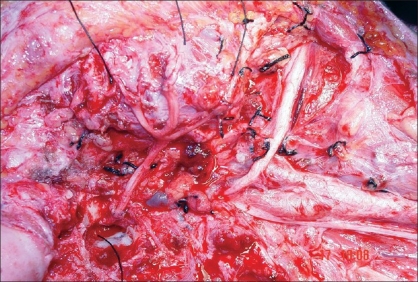
The complete nerve anastomoses are shown; the surgical field shows the distal part of the graft anastomosed to the stump of the main trunk of the facial nerve and its proximal divisions sutured to the zygomatic, buccal and mandibular branches

The postoperative period was uneventful without any complications. Topical moisturizing agents were used for postoperative eye management. Gold weight placement was recommended but not accepted by the patient. Adenoid cystic carcinoma and facial nerve invasion was confirmed in the pathological examination of the surgical specimen of the parotid gland. Four weeks after surgery, the patient was irradiated with 2 Gy fractions; the total dose was 60 Gy.

**Figure 2:**
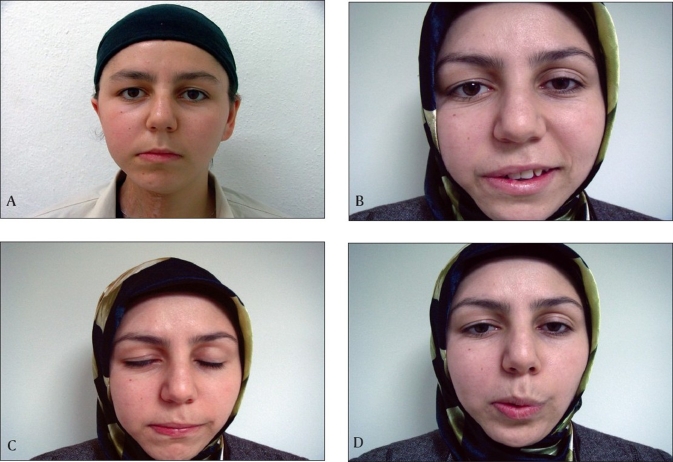
The postoperative appearance of the patient one year after surgery is shown: A) Facial symmetry at rest, B) Asymmetry with smiling, C) Eye closing, D) Whistling

The patient was followed every six months for two years. The House-Brackmann facial nerve grading system was utilized to assess postoperative facial nerve function.[12] One year after surgery, the patient achieved facial symmetry at rest, but there was still asymmetry during mimetic motion. The patient was judged to have grade IV level of facial function. Two years after operation, the patient presented with a swelling on the left side of her neck. Physical examination revealed several palpable left cervical lymph nodes ranging from 1–2 cm in diameter in the deep jugular chain. The largest one located in the upper cervical region lying in level II was excised in another medical center. The findings of the histopathological examination were consistent with inflammatory changes; no tumour cells were detected. A CT scan of the chest at this time showed a few millimetre sized nodular lesions in the lung but there was no increase in the nodular size over two years. Hence, we did not consider these lesions as evidence of metastasis; however, follow-up was done every year. At the end of four years, the patient had slightly better facial function than at the end of the first year [Figure 2].

## DISCUSSION

Malignant tumors of the parotid gland require radical resection with adequate soft tissue margins. Radical parotidectomy is advocated for the surgical treatment of high-grade malignancies and recurrent benign tumours of the parotid that intimately involve the facial nerve. Radical parotidectomy involves total extirpation of the parotid gland in conjunction with resection of the facial nerve. Sacrifice of the facial nerve is mandatory if the facial nerve is preoperatively paralyzed. Consideration should be given to grafting of the facial nerve if the proximal and distal stumps following resection of a segment of the facial nerve are available for coaptation with a nerve graft, and are histologically confirmed as uninvolved by the tumour.[11]

Facial nerve paralysis after ablative surgery of the parotid gland imposes a devastating effect on the cosmetic, functional, social, and psychological aspects of a patient's life. A multitude of surgical procedures have been described to correct the paralytic face. Cable nerve grafting has been used successfully to rehabilitate the patient after radical parotidectomy. It is an effective technique to restore voluntary facial expression and function after the sacrifice of the facial nerve for surgical resection of parotid neoplasms.[9,13] The return of facial function with cable nerve grafts is widely believed to result in superior functional and cosmetic results in comparison to cross facial nerve grafting or dynamic and static muscle slings.[14]

The two major graft sources traditionally used for extra temporal facial nerve reconstruction after radical parotidectomy include the great auricular and sural nerves. The great auricular nerve is a superficial branch of the cervical plexus, contributed to by fibres from the C2 and C3 spinal nerves. The GAN leaves the cervical plexus at the posterior border of the sternocleidomastoid muscle and courses anteriorly over the lateral surface of this muscle. It then courses superiorly, dividing into anterior and posterior branches.[15] The GAN is traced proximally until additional branches coming off the cervical nerve roots are isolated. Dissection should be done to include as much length of the graft as is possible with several branches.

We prefer to use the GAN for facial nerve reconstruction because: i) GAN is close to the operation field of parotid gland surgery, ii) Its diameter matches that of the facial nerve,[16] and iii) It has two to four proximal branches that can be anastomosed to each distal facial nerve branch. Surgical treatment of high-grade malignancies as well as advanced stage malignant tumours of the parotid preoperatively involving the facial nerve, require extended radical resection and anastomosis of each distal branch of the facial nerve. We believe that facial muscle tone and function can improve to a greater extent with the grafting of each distal facial nerve branch. The outcome obtained in this case is comparable to those reported for previous studies.[9,17]

Most ACCs arising in major salivary glands are treated surgically with the possible addition of adjunctive radiotherapy.[18] The most common indications for postoperative radiation in ACCs include advanced T-stage, positive microscopic margins, cervical nodal metastases, solid histological features, perineural invasion, and recurrent tumours.[19] These adverse prognostic indicators increase the already high risk for locoregional recurrence, distant metastases, or decreased survival that may benefit from the addition of postoperative radiation as part of an aggressive therapeutic regimen. Published reports of locoregional control for ACCs treated with surgery alone range from 40–46% five years after the surgery.

Locoregional control rates for ACCs treated by surgery along with postoperative radiation range from 64–95% five years post surgery.[3,19,20]

The effects of radiotherapy on nerve regeneration after nerve injury remain controversial. Some authors are firmly convinced that irradiation severely affects regeneration through nerve grafts, and hence, advocate early muscle transfers and static slings in lieu of nerve grafts to reconstruct the injured facial nerve. Lathrop reported that none of the patients in his series who had facial nerve grafts, demonstrated satisfactory facial function following postoperative radiation therapy.[21] Pillsbury and Fisch noted that the average return in function after radiation was only confined to restoration of symmetry at rest. When facial nerve grafting is associated with postoperative radiotherapy, it is proposed that alternative measures such as fascia lata sling and muscle transfers be used instead of facial nerve grafting.[22] On the other hand, Mc Guirt *et al.* advocated facial nerve autografting for appropriate cases of parotid malignant tumour, even if regional irradiation is to be carried out postoperatively. The benefits in their series of patients was judged to be markedly superior to the best function seen following the use of dynamic or static slings.[23] In the present case, we do not know whether irradiation affected the success of facial nerve anastomosis. However our results appear to show that it did not affect the regrowth of axons in the graft.

GAN can provide an effective graft source for reconstruction of the facial nerve and yields good functional results after radical parotidectomy.
